# Radiological and biomechanical evaluation of the ulnar nerve after the percutaneous application of the medial K-wire in the extended position on supracondylar humerus fractures: Does the postoperative long arm splint position matter?

**DOI:** 10.1097/MD.0000000000039900

**Published:** 2024-10-04

**Authors:** Mustafa Çeltik, Mustafa Hulusi Özkan, Onur Hapa, Berkay Yanik, Ali Balci, Amaç Kiray, Gülşah Zeybek, Cemre Özenbaş

**Affiliations:** a Department of Orthopedics, Ankara Oncology Training and Research Hospital, Ankara, Turkey; b Department of Orthopedics, Dokuz Eylül University Faculty of Medicine, İzmir, Turkey; c Orthopedics and Traumatology, Urla State Hospital, İzmir,Turkey; d Department of Radiology, Dokuz Eylül University Faculty of Medicine, İzmir, Turkey; e Department of Anatomy, Dokuz Eylül University Faculty of Medicine, İzmir, Turkey; f Department of Radiology, Tinaztepe University, İzmir, Turkey.

**Keywords:** elbow splint, supracondylar humerus fracture, ulnar nerve

## Abstract

Our study aimed to evaluate and compare the changes in ulnar nerve tension and strain at different elbow positions radiologically and mechanically before and after applying the medial K-wire on the supracondylar humerus fracture cadaver model. We used ten fresh frozen cadaver upper extremity specimens to measure strain and tension on the ulnar nerve in 3 different elbow positions: elbow full extension, elbow flexion–forearm supination, and elbow flexion–forearm pronation. We employed Shear wave elastography (Siemens Acuson S3000 USG, 9L4 linear probe) and a microstrain gauge (Microstrain, Inc., Burlington) to obtain our measurements. Minimum, maximum and mean stress and strain values on the nerve and its surroundings were measured and compared statistically. The mean values of elbows with full extension are statistically lower than those in elbows with 90° flexion–forearm supination and those with 90° flexion–forearm pronation positions. Statistical evaluations were performed between all of the groups. Elbow 90° flexion–forearm pronation, both minimum and maximum and mean values were statistically higher in the group, including the specimens with Kirschner applied. The mean values in the elbow full extension and elbow 90° flexion–forearm supination positions were statistically similar in the specimens with and without the K-wire applied. Despite the numerous techniques described in the literature, there is no absolute technical method to prevent ulnar nerve damage. K-wire application to the medial epicondyle with the elbow in a slightly extended position is a technique that can be applied to reduce the risk of ulnar nerve paralysis. However, it has been reported that ulnar nerve damage can be observed in cases where a splint is placed in the 90° flexion position. We hypothesize that the position of the elbow joint in the postoperative period may contribute to ulnar nerve paralysis due to soft tissue tension and strain and as a result of changing the balance of the surrounding tissues. Our findings suggest that the long arm splint applied in elbow 90° flexion and forearm pronation position should not be preferred in the postoperative period. The maximum strain values obtained in the elbow full extension were lower, suggesting that it would be appropriate to stabilize the elbow in the extension position as much as possible postoperatively.

**Level of evidence:** Level V.

## 1. Introduction

Supracondylar humerus fractures are the most common elbow fractures in children.^[1]^ Falls from height are involved in the etiology of 70% of all supracondylar humerus fractures.^[2]^ There is no consensus on which gender is more common.^[3,4]^ The preferred treatment method for these fractures is casting or fixation with K-wires after reduction. Closed reduction of displaced supracondylar humeral fractures and percutaneous Kirschner wire application was first reported by Judet in 1947 and has gained popularity over the years.^[5]^ Currently, only lateral or medial and lateral K-wire application is the preferred treatment method in such fractures. Iatrogenic nerve injury can occur at any stage of closed manipulation, percutaneous or open fixation.^[6]^ However, iatrogenic ulnar nerve injury due to the placement of the medial K-wire is considered one of the significant complications of the treatment technique for these fractures. In their study, Prashant et al^[7]^ reported a 6% iatrogenic ulnar nerve palsy rate with medial epicondyle K-wire application.

The medial K-wire application techniques described previously in the literature include the application of the K-wire while maintaining the hyperflexion of the elbow^[8]^; a small incision on the medial epicondyle to view the nerve directly^[9]^ and the use of a nerve stimulator to locate the ulnar nerve.^[10]^

The possible etiopathology that contributes to the nerve pain that develops after K-wire application in the percutaneous medial epicondyle is the position of the elbow during reduction, the position of the K-wire, its orientation and relationship with the nerve, and the angle of fixation of the post-operative joint with the splint. It is thought that there may be iatrogenic injuries due to ulnar nerve tension due to the splint applied after the procedure.

In this study, we aimed to investigate the effect of elbow joint position on ulnar nerve paralysis in the postoperative period. We hypothesized that the soft tissue tension and strain caused by the elbow joint position may change the balance of the surrounding tissues and contribute to ulnar nerve paralysis. To evaluate the dynamic anatomy of the ulnar nerve, we conducted a cadaveric study and compared the changes in ulnar nerve tension and strain in different postoperative splint positions, using ultrasonography and mechanical measurements before and after applying medial K-wire.

## 2. Materials and methods

We conducted this study on ten upper extremities from fresh frozen adult human cadavers. Approval was obtained from the Local Institutional Review Board (IRB) of Dokuz Eylul University Non-interventional Ethics Committee. It was confirmed that none of the specimens met the exclusion criteria. All specimens were free from deformities or anomalies, such as cubitus varus. Additionally, there were no limitations in joint range of motion, ulnar nerve subluxation, or elbow instability in any of the cadavers. Measurements were taken under standardized conditions, including controlled room temperature and air humidity.

The study was planned to have 2 stages (radiological and mechanical evaluation). In both stages, evaluations were made at the elbow full extension, elbow 90° flexion–forearm supination, elbow 90° flexion and forearm pronation positions, both before and after the procedure. The first stage is the radiological evaluation phase. The elastography evaluation of the ulnar nerve before the procedure on cadaver specimens was obtained with a 9L4 linear probe (4–9 MHz) in Siemens Acuson S3000 Model Ultrasonography device. Then, regions of different stiffness on and around the ulnar nerve were determined on the images obtained with the VTIQ software. The measurements were made by taking the minimum, maximum and mean values from the axonal plane on the nerve, the axonal plane around the nerve, and the longitudinal plane of the nerve. Measurements for these areas were repeatedly made in full extension, 90° flexion-supination, and 90° flexion-pronation positions. The minimum, maximum and average values were recorded. Multiple measurements from these areas measured elasticity values in meters/second. Since the ultrasonographic measurements made in one of our cadavers were not found consistent by the radiologist, it was excluded from the statistics.

After the initial evaluation, a 2 mm medial K-wire was applied to the elbow in the full extension. The position of the K-wire and its relation to the ulnar nerve was confirmed by X-ray and USG.

**Figure 1.**
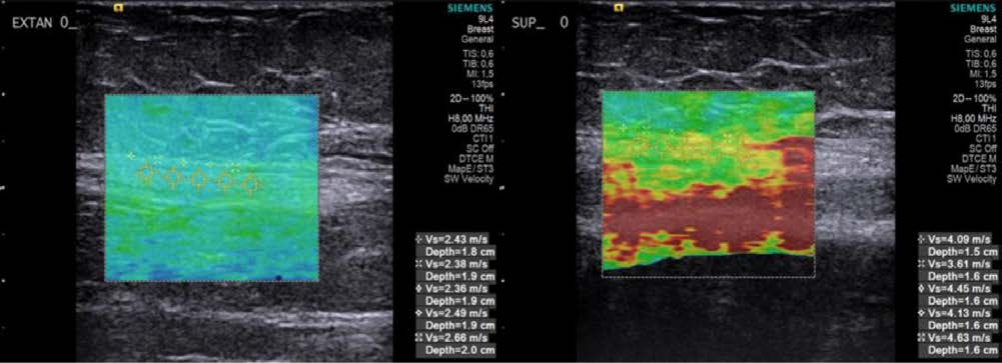
Shear wave elastography (USG) findings in different positions (longitudinal plane, the measurements were made on the nerve. The image on the left shows the USG readings from an elbow in an extended position. The image on the left shows the USG readings from an elbow in 90° flexion and a forearm in a supination position.

The measurements made in the previous step were repeated to evaluate the effect of K-wire application on elastograms taken around the ulnar nerve. The measurements were made on the axonal plane of the nerve, the axonal plane around the nerve, and the longitudinal plane of the nerve. Measurements for these areas were repeatedly made in full extension, 90° flexion-supination, and 90° flexion-pronation positions (Fig. 1).

The minimum, maximum and average values were recorded. Multiple measurements from these areas measured elasticity values in meters/second.

After the radiological assessment, the upper extremity specimens were transported to the anatomy laboratory for the second stage of the study. A 5 cm long skin incision centered on the medial epicondyle was made. After dissection from the subcutaneous tissue, the ulnar nerve was identified. A microstrain gauge was inserted directly into the ulnar nerve proximal to the medial epicondyle with contact differential variable reluctance transducer sensors (Microstrain, Inc, Burlington). The microtension meter makes its measurements by producing a voltage change with the movements of the barbs of its coils.

**Figure 2.**
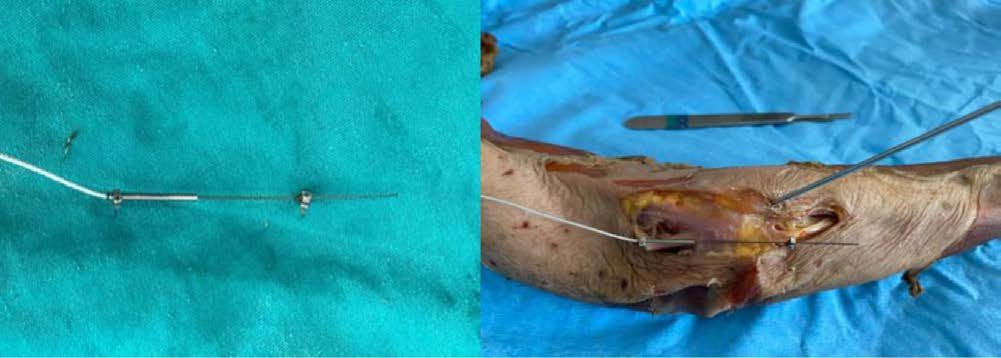
The root and moving parts of the DVRT sensor were fixed to the nerve with the help of 2 sharp pins, and measurements were made before and after the K-wire. DVRT = differential variable reluctance transducer.

DVRT sensor data was started from position 0 for each cadaver at the beginning of movement. All movement data were taken from a single channel, 0 to 3 mm, with an accuracy of 1 μm, from a single channel, including the K-wired and non-K-wired specimens. The elongation data obtained were converted to Excel data, and the voltage values in each movement were converted into (mm). Thus, maximum elongation and movement change data were collected for each cadaver (Fig. 2).

The K-wire was pulled out after recording the measurements in different positions. The measurements were repeated and recorded for the same positions where the K-wire was absent in the cadavers. Mechanical measurements evaluated the effect of K-wire withdrawal on the nerve.

Data were evaluated in the statistical package program of IBM SPSS Statistics Standard Concurrent User V 26 (IBM Corp., Armonk). Descriptive statistics were given as a number of units (n), percent (%), mean (x ®), and standard error (SE). Since measurements for all variables in the study were taken from the same cadavers, statistical comparisons were made with mixed-effect linear models, including fixed and random effects. Bonferroni correction was applied to compare the main effects. A *P* value of < .05 was considered statistically significant.

## 3. Results

Measurements obtained from the *nerve in the axial plane* between groups are shown in Table 1. The values measured in 3 different elbow position positions were statistically similar in cadavers with and without the K-wire. According to in-group comparisons using the measurements in the *axial* plane on cadavers without K-wire, the elbow full extension values are statistically lower than the other 2 positions. Measurements obtained from the cadavers with K-wire, the differences between the values in different positions are not statistically significant.

**Table 1:** Valuations: statistical comparison of ulnar nerve tension variation in different elbow positions. Measurements are obtained in an axial plane, from the ulnar nerve itself.

	Measurements				Test statistics*	
	Cadavers without the medial K-wire		Cadavers with the medial K-wire			
	Average	SE	Average	SE	*F*	*P*
Axial plane-nerve						
Full extension	2.974‡	0.190	2.934	0.276	0.027	0.874
90° flexion-supination	3.732‡	0.244	3.211	0.283	2.328	0.166
90° flexion-pronation	3.540‡	0.271	3.439	0.323	0.192	0.673
Test statistics†	*F* = 10.432; *P* = **.006**		*F* = 1.227; *P* = .343			

SE = standard error.

* Cross-trade comparisons in each position.

† Comparisons between positions in each trade type.

‡ Indicates differences in within-group comparisons. Position values with the same letters are statistically similar.

According to the comparisons using the measurements obtained from the *nerve surroundings in the axial plane* between groups in Table 2, the values measured in 3 different elbow positions were statistically similar in cadavers with and without the K-wire. According to in-group comparisons using the measurements in the *axial* plane on cadavers without K-wire, the elbow full extension values are statistically lower than the elbow 90° flexion–forearm supination position. Measurements obtained from the cadavers with K-wire, the differences between the values in different positions are not statistically significant.

**Table 2:** Statistical valuations: statistical comparison of ulnar nerve tension variation in different elbow positions. Measurements are obtained from the axial plane and the area surrounding the nerve.

	Measurements				Test statistics*	
	Cadavers without the medial K-wire		Cadavers with the medial K-wire			
	Average	SE	Average	SE	*F*	*P*
Axial plane nerve surroundings: mean						
Full extension	2.527‡	0.104	2.366	0.096	4.368	.070
90° flexion-supination	3.139‡	0.169	2.897	0.210	2.961	.125
90° flexion-pronation	2.864‡	0.163	2.793	0.198	0.226	.647
Test statistics†	*F* = 5.275; *P* = **.035**		*F* = 4.407; *P* = .057			

SE = standard error.

* Cross-trade comparisons in each position.

† Comparisons between positions in each trade type.

‡ ‡Indicates differences in within-group comparisons. Position values with the same letters are statistically similar.

According to the comparisons using the measurements obtained from the *nerve in* the *longitudinal plane* between groups in Table 3, the minimum values of elbow full extension and elbow 90° flexion–forearm supination were statistically similar in the cadavers with and without the medial K-wire. The minimum values obtained in the elbow 90° flexion–forearm pronation position were statistically higher in the K-wire applied procedure group.

**Table 3:** Statistical valuations: statistical comparison of ulnar nerve tension variation in different elbow positions. The measurements are obtained for the longitudinal plane. Minimum values were compared.

	Measurements				Test statistics*	
	Cadavers without the medial K-wire		Cadavers with the medial K-wire			
	Average	SE	Average	SE	*F*	*P*
Longitudinal plane: minimum						
Full extension	2.737‡	0.196	2.640‡	0.199	0.258	.625
90° flexion-supination	3.352‡	0.246	3.170‡	0.229	0.964	.355
90° flexion-pronation	3.103‡	0.264	3.689‡	0.236	6.827	**.031**
Test statistics†	*F* = 4.463; *P* = **.049**		*F* = 6.497; *P* = **.021**			

SE = standard error.

* Cross-trade comparisons in each position.

† Comparisons between positions in each trade type.

‡ Indicates differences in within-group comparisons. Position values with the same letters are statistically similar.

According to in-group comparisons using the measurements in the longitudinal plane, the *minimum* values of elbow full extension in the groups without K-wire were statistically lower than the minimum values of elbow 90° flexion–forearm supination. In the measurements made on the cadavers with the K-wire applied, the minimum values of elbow full extension are statistically lower than those measured in the elbow 90° flexion–forearm pronation position.

According to the comparisons in Table 4 between the groups using the measurements in the *longitudinal plane*, the *maximum* values obtained elbow full extension and elbow 90° flexion–forearm supination positions were statistically similar in the cadavers with and without K-wire. The maximum values of elbow 90° flexion–forearm pronation were statistically higher in the cadavers with the medial K-wire applied.

**Table 4:** Statistical valuations: statistical comparison of ulnar nerve tension variation in different elbow positions. The measurements were obtained from the longitudinal plane and the maximum values were compared.

	Measurements				Test statistics*	
	Cadavers without the medial K-wire		Cadavers with the medial K-wire			
	Average	SE	Average	SE	*F*	*P*
Longitudinal plane: maximum						
Full extension	3.194‡	0.220	3.131‡	0.230	0.097	.763
90° flexion-supination	4.377‡	0.287	4.422‡	0.412	0.030	.867
90° flexion-pronation	3.850‡	0.314	4.631‡	0.277	8.548	**.019**
Test statistics†	*F* = 8.929; *P* = **.009**		*F* = 15.025; *P* = **.002**			

SE = standard error.

* Cross-trade comparisons in each position.

† Comparisons between positions in each trade type.

‡ Indicates differences in within-group comparisons. Position values with the same letters are statistically similar.

According to in-group comparisons using the measurements in the longitudinal plane, the *maximum* values in the elbow full extension position were statistically lower than the maximum values of 90° flexion of the elbow and supination of the forearm of the cadavers without the medial K-wire. The *maximum* values of the elbow in full extension were statistically lower than the maximum values of the other 2 positions on the cadavers with the K-wire.

According to the comparisons in Table 5 between the groups using the measurements in the *longitudinal plane*, the *mean* values obtained for elbow full extension and elbow 90° flexion–forearm supination positions were statistically similar in the cadavers with and without K-wire. The mean values of elbow 90° flexion–forearm pronation were statistically higher in the cadavers with the medial K-wire applied.

**Table 5:** Statistical valuations: statistical comparison of ulnar nerve tension variation in different elbow positions. Measurements were obtained from the longitudinal plane, and the mean tension values were compared.

	Measurements				Test statistics*	
	Cadavers without the medial K-wire		Cadavers with the medial K-wire			
	Average	SE	Average	SE	*F*	*P*
Longitudinal plane: mean						
Full extension	2.946‡	0.207	2.894‡	0.202	0.078	.787
90° flexion-supination	3.753‡	0.283	3.814‡	0.308	0.083	.781
90° flexion-pronation	3.430‡	0.290	4.196‡	0.246	9.794	**.014**
Test statistics†	*F* = 6.017; *P* = **.025**		*F* = 11.983; *P* = **.004**			

SE = standard error.

* Cross-trade comparisons in each position.

† Comparisons between positions in each trade type.

‡ Indicates differences in within-group comparisons. Position values with the same letters are statistically similar.

According to in-group comparisons, the *mean* values obtained for elbow full extension are statistically lower than those of the elbow 90° flexion–forearm supination position in the group without the K-wire. The *mean* values obtained from the elbow in full extension were statistically lower than the maximum values of the other 2 positions on the cadavers with the K-wire.

Measurements recorded using the Microstrain DVRT sensor were summarized in Table 6. According to the comparisons between the groups in Table 6, the values obtained in the elbow full extension, elbow 90° flexion–forearm supination, and elbow 90° flexion–forearm pronation positions were statistically similar in the cadaver groups with and without K-wire.

**Table 6:** Statistical valuations: statistical comparison of ulnar nerve tension variation in different elbow positions. Strain values were obtained using DVTR sensors.

	Measurements				Test statistics*	
	Cadavers without the medial K-wire		Cadavers with the medial K-wire			
	Average	SE	Average	SE	*F*	*P*
DVRT sensor data Length: strain						
Full extension	0.0	0.0	0.0	0.0	–	–
Flexion-supination	2.011‡	0.286	1.976	0.485‡	0.006	.941
Flexion-pronation	2.297‡	0.449	2.162	0.579‡	0.123	.734
Again full extension	0.144‡	0.094	−0.106	0.106‡	3.747	.085
Test statistics†	*F* = 31.354; *P* < **.001**		*F* = 9.060; *P* = **.007**			

SE = standard error.

* Cross-trade comparisons in each position.

† Comparisons between positions in each trade type.

‡ Indicates differences in within-group comparisons. Position values with the same letters are statistically similar.

According to in-group comparisons, the elbow 90° flexion–forearm supination and elbow 90° flexion–forearm pronation length values were statistically higher than the elbow full extension values in both K-wire and non-K-wired cadavers.

In summary, the study found that the nerve strain was lowest in the full extension position, both in cadavers with and without K-wire, even though there was no statistically significant difference in nerve strain between the groups in the other positions measured by the Microstrain DVRT sensor.

## 4. Discussion

In our study, we collected radiological and mechanical data to evaluate the tension and strain of the ulnar nerve at elbow full extension, elbow 90° flexion–forearm supination, and elbow 90° flexion–forearm pronation positions before and after K-wire application. The tension of the ulnar nerve gradually increases to the elbow flexion position, even without applying the K-wire, in the evaluations made with upper extremity cadaver specimens. We evaluated the data using minimum, maximum, and average values. In the measurements made on the nerve in the longitudinal plane on the K-wire-applied pieces, the mean values of elbow full extension were statistically lower than those of elbow 90° flexion–forearm supination and elbow 90° flexion–forearm pronation. In the statistical evaluation between all groups, the minimum, maximum, and mean values of elbow 90° flexion–forearm pronation were statistically higher in the K-wire group. Therefore, the least preferred splint position in the postoperative period is the 90° elbow flexion–forearm pronation position. Elbow full extension and elbow 90° flexion–forearm supination mean values were statistically similar in groups with and without K-wire applied.

Although there was no statistically significant difference between the groups in the nerve strain measured by the Microstrain DVRT sensor depending on the positions, it is a statistically significant finding that the nerve strain is at the lowest level in the full extension position in the evaluations made both in the cadavers with the K-wire applied and the cadavers without the K-wire.

Our study tried to reveal whether the elbow splint position after medial K-wire application contributed to ulnar nerve injury, one of the most important complications of the surgical treatment of supracondylar humerus fractures. Iatrogenic ulnar nerve injury is the most common complication after surgery for supracondylar humerus fractures,^[11,12]^ and the incidence reported in the literature is between 1.4% and 15%.^[9,13–15]^ Flexion-type supracondylar humerus fractures have a higher incidence of ulnar nerve entrapment, more frequently necessitating open reduction and internal fixation.^[16]^

The literature discusses the application of the medial wire extensively. The study by Gupta et al^[17]^ found that placing the medial K-wire first, followed by the lateral K-wire, in treating supracondylar humerus fractures in children via closed reduction resulted in stable fixation and favorable clinical outcomes. Whether applied first or after the application of the lateral K-wire, the medial K-wire applied to increase stability should be applied carefully in terms of causing iatrogenic ulnar nerve damage.

In the literature, temporary motor or sensory changes often occur after the nerve compression that develops after the direct penetration of the ulnar nerve with the K-wire during fixation or the fixation of the surrounding tissues by the K-wire. Still, the prognosis of the losses cannot be predicted. In most patients with ulnar nerve damage, return to full function and complete disappearance of symptoms can be observed within weeks to months.^[13,14,18,19]^

Ulnar nerve stiffness is evaluated radiologically by the Shear Wave Elastography method and mechanically by a Microstrain DVRT sensor before and after applying the medial K-wire. All the measurements were elbow full extension, elbow 90° flexion–forearm supination and elbow 90° flexion–forearm pronation analyzed separately with their movements. The nerves are naturally under tension due to the soft tissues surrounding them during elbow movements.^[20]^ Under higher tension, nerve fascicles become tighter,^[21]^ increasing Shear Wave Elastogram values. Therefore, if the elastogram velocity values are high, it can be concluded that the tension of the nerve increases.

Prashant et al’s study on 62 patients with Gartland type III supracondylar fractures compared only the lateral wire and lateral and medial cross-wire application. Medial wire application in his studies: After the relative stabilization was achieved after the lateral wire, it was determined that the elbow was placed in the extension position under 90°, and the ulnar nerve was rolled backwards with the finger. In the study, which reported that the long arm was splinted with the extremity in 90° flexion after the operation, it is noteworthy that 2 patients (6.5%) in the medial-lateral access group had iatrogenic ulnar nerve palsy^[7]^

According to a study by Sinikumpu et al,^[22]^ 16% of the cases with extension-type supracondylar humerus fractures experienced late-onset ulnar nerve-related symptoms such as hypersensitivity after a mean of 12 years of follow-up. The study also suggests these symptoms may be associated with local deformity and changing load-bearing angle or soft tissue thickening around the nerve after trauma.^[22]^ The study suggests that soft tissue problems in the postoperative period may contribute to ulnar nerve functions.

De Pellegrin et al^[23]^ compared the advantages of supine and prone positions in the surgical treatment of supracondylar humerus fractures. They stated that iatrogenic nerve injury is less common in the prone position. They attributed this to the hypermobility of the ulnar nerve in the cubital tunnel in children^[24]^ and its tendency to subluxate anteriorly over the medial epicondyle, especially when the elbow is hyperflexed. Therefore, they explained that the nerve will be more risky in the supine position.^[23]^

During percutaneous medial K-wire application, it is recommended to apply the K-wire in the slightly extended position to prevent ulnar nerve paralysis.^[25]^ Georgiadis et al^[26]^ reported that 215 medial K-wires were applied to patients with pediatric supracondylar humerus fractures in a maximum extension position of 60°, and no ulnar nerve involvement was observed after an average follow-up period of 13 weeks. In this study, they stated that after the relative stabilization of the lateral input wires, partial elbow extension and glenohumeral external rotation can prevent ulnar nerve involvement.

There are studies indicating that the risk of iatrogenic ulnar nerve injury can be reduced with the mini-open medial incision method.^[9]^ The literature shows that the risk of ulnar nerve involvement can be reduced by placing the elbow in a slightly extended position while applying the K-wire to the medial epicondyle.^[25]^ There are also studies indicating that iatrogenic ulnar nerve injuries associated with medial wire fixation are resolved after nerve exploration^[27]^ and medial wire entry site replacement.^[9,28]^ In contrast, some studies also suggest nerve damage typically occurs during wire placement; thus, early wire removal does not significantly improve outcomes.^[29]^

In the study by Kocher et al, only the lateral wired method and medial and lateral wired applications were compared in mild elbow extension position after exploration with a small medial incision. They reported no cases of iatrogenic ulnar nerve injury in either group during their follow-up.^[30]^ They reported in their study that the risk of iatrogenic ulnar nerve injury can be reduced by elbow extension during medial wire placement.

A comparative study by Erçin et al^[31]^ found that the medial mini-open technique had results similar to the percutaneous technique and had an equal risk of iatrogenic ulnar nerve damage. However, there are cases in the literature showing that the medial mini-open technique also has cosmetic disadvantages^[32]^

In a study by Belhan et al, they investigated the dynamic and morphological changes of the ulnar nerve in the cubital tunnel of medial K-wire application in supracondylar humerus fractures. They put forward the thesis that the K-wire applied in the hyperflexion position fixes the cubital tunnel retinaculum in the tense position. Accordingly, when the elbow is extended, the retinaculum will be stretched further due to the tension of the nerve. Their study reported that the dynamic and morphological changes of the ulnar nerve in the cubital tunnel were not caused by the fracture itself but rather by applying the medial K-wire.^[33]^

In the study of Eidelman et al with 91 patients with supracondylar humerus fractures, the medial wire was applied when the elbow was in full extension, and the elbow was placed in 90° of flexion and forearm pronation in the postoperative period. It has been shown that ulnar nerve palsy was not observed in any of the patients they followed up with in this way.^[25]^

Woo et al^[34]^ reported that ulnar neuropathy was not observed in any patient after percutaneous medial wire application with the elbow not in more than 45° flexion in their study on 125 patients.

In a retrospective study conducted by Kwak-Lee et al with 291 patients, it was reported that the application of the medial K-wire was applied in the elbow extension position to reduce the possibility of anterior subluxation of the ulnar nerve on the medial epicondyle. In the postoperative period, it has been reported that after initially applying a temporary splint with the elbow in 70°of flexion, the patient was converted to a 90° flexion long arm cast at the first postoperative visit. It was stated that iatrogenic nerve injury was not observed both in cases where only lateral K-wire was applied and in cases where lateral and medial K-wire was applied.^[35]^

Although many methods mentioned above have been mentioned in the literature to prevent ulnar nerve paralysis, there is no consensus on a definitive treatment technique for the absolute prevention of ulnar nerve paralysis in the treatment algorithm.

This study concludes that the long arm splint applied in elbow 90° flexion and forearm pronation position should not be preferred in the postoperative period. The study found that the maximum strain values obtained in the elbow full extension were lower, suggesting that it would be appropriate to stabilize the elbow in the extension position as much as possible postoperatively.

This study has some limitations. The main limitation is that the experiment was conducted on adult cadavers, while K-wires are typically used to treat pediatric patients. The ethical dilemmas and limited availability of pediatric cadavers necessitated the use of adult upper extremity specimens. Although examining nerve samples in fresh cadavers is a well-established protocol, laboratory results may not reflect the in vivo situation. Additionally, since this is a cadaveric study, a clinical relationship cannot be established clearly regarding ulnar nerve tension and the degree of symptoms related to strain. Because some dissection around the nerve is required to perform strain measurements, some properties of the nerve and surrounding soft tissues may differ from the in vivo situation, even if performed on freshly frozen cadavers. Further experimental and clinical studies are needed to evaluate the effects of medial K-wire application on ulnar nerve iatrogenic effects.

## Author contributions

**Conceptualization:** Gülşah Zeybek.

**Data curation:** Mustafa Çeltik, Cemre Özenbaş.

**Formal analysis:** Mustafa Hulusi Özkan, Cemre Özenbaş.

**Investigation:** Mustafa Hulusi Özkan, Ali Balci, Amaç Kiray, Gülşah Zeybek, Cemre Özenbaş.

**Methodology:** Onur Hapa, Berkay Yanik, Ali Balci, Gülşah Zeybek.

**Project administration:** Berkay Yanik, Gülşah Zeybek.

**Supervision:** Amaç Kiray.

**Writing – original draft:** Mustafa Çeltik, Cemre Özenbaş.

**Writing – review & editing:** Onur Hapa, Cemre Özenbaş.
